# Roux-en-Y Gastric Bypass Surgery in the Management of Familial Partial Lipodystrophy Type 1

**DOI:** 10.1210/jc.2017-01235

**Published:** 2017-07-26

**Authors:** Audrey Melvin, Claire Adams, Catherine Flanagan, Lisa Gaff, Barbara Gratton, Fiona Gribble, Geoffrey Roberts, Robert K. Semple, Stephen O’Rahilly, Francesco Rubino, Anna Stears, David B. Savage

**Affiliations:** 1 National Severe Insulin Resistance Service, Addenbrooke’s Hospital, Cambridge CB2 0QQ, United Kingdom; 2 Metabolic Research Laboratories, Wellcome Trust-Medical Research Council Institute of Metabolic Science, University of Cambridge, Cambridge CB2 0QQ, United Kingdom; 3 Bariatric and Metabolic Surgery, Division of Diabetes and Nutritional Sciences, King’s College London, London SE5 9NU, United Kingdom

## Abstract

**Context:**

Familial partial lipodystrophy type 1 (FPLD1) is an extreme form of central adiposity, with peripheral lipodystrophy associated with severe manifestations of the metabolic syndrome, often poorly responsive to standard therapeutic approaches. Body mass index in FPLD1 varies but, in many cases, is below the level at which metabolic surgery is usually considered as a therapeutic option.

**Design:**

We detailed the metabolic response to gastric bypass surgery of three patients with FPLD1, refractory to medical therapy.

**Results:**

Roux-en-Y gastric bypass (RYGB) was associated with weight loss and substantial improvements in glycemic control and insulin sensitivity. All three patients were able to stop using insulin. Glucose tolerance testing in one patient demonstrated an increase in L-cell–derived gut hormone responses postoperatively.

**Conclusion:**

RYGB surgery substantially improved glycemic control in three patients with FPLD1, two of whom had body mass indices below 30 kg/m^2^. RYGB should be considered in patients with partial lipodystrophy and refractory metabolic disease.

## Teaser

We studied the clinical response of three patients with familial partial lipodystrophy type 1 and refractory diabetes to RYGB surgery and documented dramatic clinical improvements.

Familial partial lipodystrophy type 1 (FPLD1) is characterized by central fat accumulation, reduced peripheral subcutaneous adipose tissue, and ectopic fat deposition (1). The metabolic sequelae of FPLD1 can be severe and include insulin-resistant type 2 diabetes, dyslipidemia, nonalcoholic fatty liver disease (NAFLD), and hyperandrogenism. Patients with FPLD1 have long posed a treatment challenge, with current approaches targeting metabolic complications of ectopic fat deposition using similar strategies to those used in obese patients with diabetes (2). Recombinant leptin therapy is very effective in patients with generalized lipodystrophy and to a lesser extent in other forms of partial lipodystrophy, but leptin levels in most patients with FPLD1 are well above current thresholds (4 to 10 µg/L) for leptin therapy (3). Here we report on the metabolic outcomes of gastric bypass surgery in three patients with FPLD1.

## Materials and Methods

Participants were assessed at the National Severe Insulin Resistance Service, Cambridge University Hospital, and further evaluated at the National Institute for Health Research/Wellcome Trust Clinical Research Facility. Ethical approval was obtained from the East of England-Cambridge Research Ethics Committee, and participants have consented to the use of data for scientific publication. Dual-energy X-ray absorptiometry (DEXA) imaging was undertaken on Lunar iDXA or Lunar Prodigy (GE Health Care), with specialized assays completed at the Core Biochemical Assay Laboratory, University of Cambridge.

## Results

### Patient A

**Figure 1.**
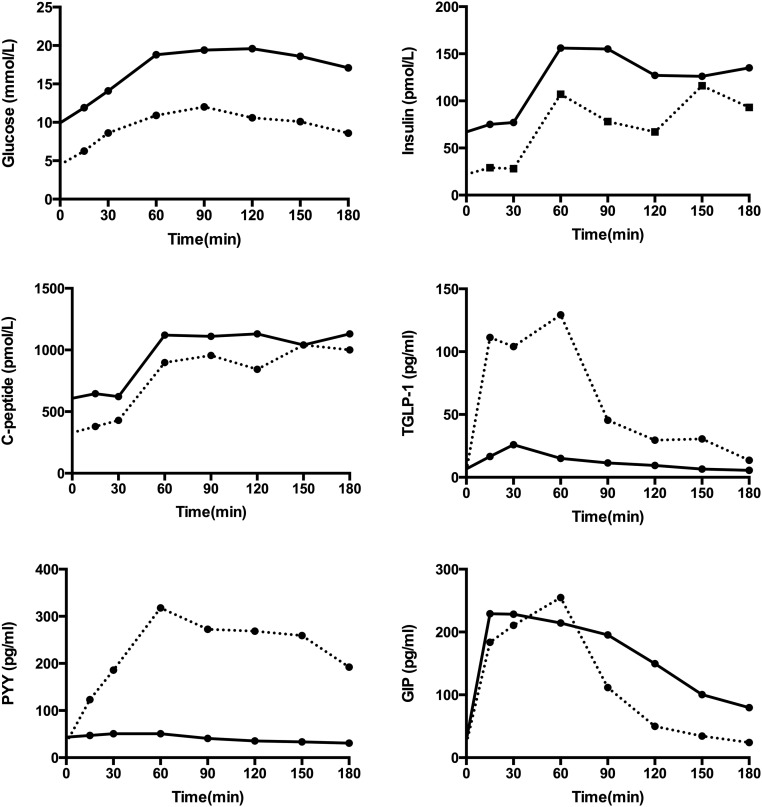
The pancreatic and enteroendocrine hormonal responses of patient A to a 50-g oral glucose tolerance test preoperatively (black line) and 3 months following RYGB surgery (dashed line). Glucose tolerance improved postoperatively. GLP-1 and PYY response to glucose load increased at 3 months, while glucose-dependent insulinotropic polypeptide was largely unchanged.

A 49-year-old female attending the National Severe Insulin Resistance Service had originally presented at age 32 years with gestational diabetes, which persisted postpartum. She developed central adiposity during puberty. She has a positive family history for diabetes (Supplemental Fig. 1). Prior to referral, her glycemia had been managed with metformin (stopped due to gastrointestinal intolerance) and gliclazide, followed by insulin therapy and exenatide. Examination and DEXA imaging (Supplemental Fig. 2) confirmed central adiposity with peripheral lipodystrophy. Biochemical tests were consistent with those typical of patients with partial lipodystrophy. Therefore, having excluded mutations in *LMNA* (lamin A/C) and *PPARG* (peroxisone proliferator activated receptor *γ*), a diagnosis of FPLD1 was made. A low-calorie diet was introduced, but glycemic control remained suboptimal despite large insulin doses. Triglyceride levels were modestly elevated (>2 mmol/L) (Table 1). Referral for gastric bypass surgery was made at the patient’s request on clinical grounds at age 53. At this time, her body mass index (BMI) was 27.4 kg/m^2^. Blood glucose readings fell within days of surgery, requiring the cessation of all medication (Supplemental Fig. 3), with a corresponding fall in hemoglobin A_1c_. Oral glucose tolerance testing was undertaken before and 3 months after surgery (Fig. 1). Postoperatively, glucose tolerance was greatly improved, and this was associated with dramatically increased L-cell–derived satiety hormones glucagonlike peptide-1 (GLP-1) and peptide YY (PYY). Glucose-dependent insulinotropic polypeptide levels were unchanged. Consistent with the reduction in adiposity and fasting glucose, fasting insulin levels were markedly reduced, whereas the incremental insulin response to the glucose load was similar to that found preoperatively, despite the much lower postprandial glucose levels.

**Table 1.** **Impact of RYGB on Anthropometric and Biochemical Measurements**

	**Patient A**		**Patient B**			**Patient C**		
	**Preoperative**	**Postoperative 3 Months**	**Preoperative**	**Postoperative 3 Months**	**Postoperative 40 Months**	**Preoperative**	**Postoperative 18 Months**	**Ref Range**
BMI (kg/m^2^)	27.4	21.7	32.4	24.7	26.8	29.7	22.9	
Body fat (%)*^a^*	49.1	42.9	37	24.5	NA	NA	28	
Android:gynoid ratio	1.46	1.28	1.53	1.38	NA	NA	1.16	
Fat mass ratio*^b^*	1.63	1.56	1.80	1.83	NA	NA	1.45	
Hepatic fat fraction (%)*^c^*	7	7	6	3	3.2	20	3.2	
HbA1_c_ (mmol/mol)	75	54	118	50	47	113	48	36–45
HbA1_c_ (%)	9.0	7.1	12.9	6.7	6.3	12.5	6.5	4.9–6.3
Leptin (ng/mL)	59.1	7.6	15.6	3	9.8	20.9	6.2	*^d^*
Adiponectin (g/mL)	5.9	8	3.8	4.6	7.1	4.3	16.5	*^e^*
Leptin/adiponectin ratio (ng/µg)	10.0	1.0	4.1	0.7	1.3	4.9	0.4	
Insulin (pmol/L)	67	18	67	35	29	132	33	0–80
C-peptide (pmol/L)	608	312	890	662	774	1897	1210	170–960
Cholesterol (mmol/L)	8.2	7.6	4.9	2.6	3.7	5.3	4.1	
Low-density lipoprotein (mmol/L)	6.3	5.5	3.4	1.4	1.8	2.6	1.8	
Triglyceride (mmol/L)	2	2.8	1.1	0.5	0.7	3.5	1.9	
High-density lipoprotein (mmol/L)	1.0	0.8	1.0	1.0	1.5	1.1	1.4	
Albumin (g/L)	37	34	38	38	37	38	35	35–50
Bilirubin (umol/L)	11	8	6	6	6	5	3	0–20
Alk phos (U/L)	106	111	126	72	47	132	96	30–130
Alanine aminotransferase (U/L)	18	18	27	32	26	51	22	7–40
GGT (U/L)	21	11	28	19	10	66	11	0–37
TSH (mU/L)	2.3	2.5	1.4	1	1.3	1.0	1.2	0.35–5.55
FT_4_ (pmol/L)	14.2	NA	15.8	12.8	11.5	15.3	10.8	10.0–19.8
Insulin dose (unit/d)	110–200	0	80–120	0	0	20–40	0	
Additional medical therapy	Telmisartan		Metformin	Metformin	Metformin	Metformin		
			Sitagliptin	Simvastatin	Simvastatin	Gliclazide		
			Simvastatin			Liraglutide		
			Ramipril			Losartan		

Abbreviations: Alk phos, alkaline phophatase; FT_4_, free T_4_; GGT, gamma-glutamyl transferase; HbA_1c_, hemoglobin A_1c_; NA, not available; TSH, thyroid-stimulating hormone.

^a^ DEXA.

^b^ Fat mass ratio represents percentage trunk fat compared with legs.

^c^ Magnetic resonance imaging.

^d^ Leptin ref range (ng/mL) (female): BMI 25 kg/m^2^ = 2.5 to 24.4; BMI 25 to 30 kg/m^2^ = 8.6 to 38.9; BMI 30 to 35 kg/m^2^ = 14.9 to 60.9; BMI >35 kg/m^2^ = 27.7 to 113.6.

^e^ Adiponectin ref range (µg/mL) (female): BMI 25 kg/m^2^ = 4.4 to 17.7; BMI 25 to 30 kg/m^2^ = 3.5 to 15.5; BMI 30 to 35 kg/m^2^ = 2.6 to 14.9; BMI >35 kg/m^2^ = 2.6 to 17.1.

### Patients B and C

Two further female patients were diagnosed with FPLD1 following the exclusion of hypercortisolism and pathogenic *PPARG* variants. Both displayed the classical clinical features of FPLD1. Patient B had hypertension, dyslipidemia, NAFLD, and secondary diabetes requiring >180 units of insulin daily. She gained weight in parallel with increasing insulin doses. A structured weight management program achieved modest weight loss, but HbA_1c_ remained >100 mmol/mol (11.3%), and retinopathy and nephropathy had developed. Patient C also experienced NAFLD, dyslipidemia, and poorly controlled diabetes despite multiple glucose-lowering therapies. She did respond favorably (weight loss of 4.6 kg and improved glycemic control) to a low-energy liquid diet but was unable to sustain the weight loss. Both patients were referred for Roux-en-Y gastric bypass (RYGB) surgery. Patient B was age 44 and obese with a BMI of 32.4 kg/m^2^. Postoperatively, her insulin was stopped due to dramatic improvements in blood glucose, and she is currently well controlled on metformin alone. At age 53 with a BMI of 29.7 kg/m^2^, patient C underwent gastric bypass surgery that resulted in a dramatic improvement in diabetes control and cessation of all glucose-lowering medication. In addition to weight loss, both patients experienced a reduction in hepatic fat and fasting insulin levels (Table 1). Patient B had an uncomplicated course and remains well 40 months post RYGB. Patient C developed a gastric ulcer at 18 months postoperatively and reported dental caries, but she too remains metabolically well.

## Discussion

FPLD1 is a disorder characterized by reduced peripheral adipose tissue expandability (1). This is thought to engender a mismatch between the need and capacity to buffer excess energy intake leading to insulin resistance and its associated metabolic problems. The mainstay of therapy, although challenging to achieve, is reduced caloric intake (Supplemental Fig. 4) (2). Patients with FPLD1 who lose weight manifest significant metabolic improvements. However, they struggle to sustain this, perhaps in part due to relative leptin deficiency secondary to peripheral lipodystrophy. RYGB, previously viewed solely as a bariatric procedure, has emerged as effective therapy in the management of metabolic dysfunction in obese individuals, raising the question of its role in nonobese individuals with deranged glycemic control.

**Figure 2.**
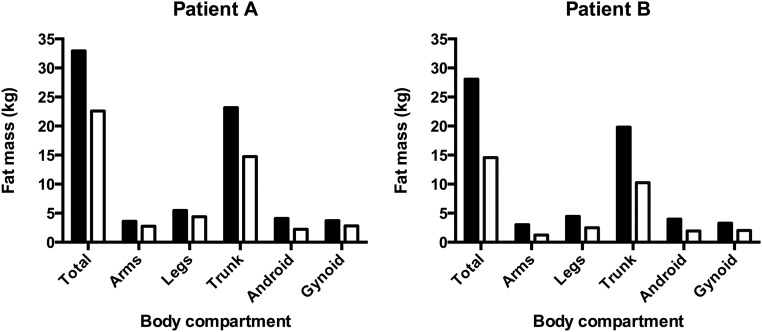
The changes in fat mass between the preoperative (black bars) and 3-month postoperative (white bars) periods in two patients with FPLD1 undergoing RYGB.

We assessed the impact of RYGB on fat mass in three patients with FPLD1; all manifested substantial sustained weight loss, and DEXA imaging demonstrated a reduction in fat across all body compartments. A marked decline in android fat mass was evident, owing to the pathological distribution of fat observed in FLPD1 (Fig. 2). Our patients reported reduced hunger postoperatively, despite the postoperative drop in leptin levels, and it is likely that this is the primary reason for the beneficial impact of surgery on energy balance. The precise basis for the appetitive changes that occur post RYGB surgery in all patient groups is a topic of intense scientific activity, but the increased postprandial levels of GLP-1 and PYY3 through PPY36, hormones with an anorectic effect, are likely to play an important role.

The most dramatic clinical impact in all three patients was on the poorly controlled type 2 diabetes that was present preoperatively. The improvement in glycemic control is consistent with that seen in obese individuals with type 2 diabetes who undergo bariatric surgery (4) or who sustain a period of negative energy balance due to intense dietary restriction (5). Patients with FPLD1 are frequently very insulin resistant, almost certainly due to lipotoxicity in insulin-responsive tissues such as muscle and liver, which are responsible for a large component of glucose clearance; this was reflected in the high insulin doses and poor glycemic control of patients A and B and in the high fasting insulin levels in patient C. Measures of insulin resistance (fasting insulin and leptin-adiponectin ratio) were markedly improved in all three patients postoperatively, likely due to the reduction in lipotoxic load to insulin-responsive tissues as a result of sustained negative energy balance. It is possible that the increased GLP-1 levels had an additional impact on glycemic control through their incretin effect, but the dramatically different fasting and postload glucose levels that were present pre- and postoperatively in patient A make it impossible to comment meaningfully on this.

Patient A did not have a marked elevation in hepatic fat (at least as measured noninvasively) or in circulating triglycerides, but these measures were more markedly abnormal in patients B and C in whom the beneficial impact of surgery on liver fat and circulating lipids was more obvious.

All of our patients have remained clinically well at follow-up; however, patient C developed a gastric ulcer, which was treated effectively with medical therapy. Although serious adverse events are uncommon following metabolic procedures, patients should be counseled on the procedure risks and the requirement for extended follow-up and nutritional supplementation. To date, RYGB has been the only metabolic surgery reported in patients with lipodystrophy, and it is unknown if similar efficacy would be seen with other weight-lowering procedures.

## Conclusion

Reports of the use of RYGB in the management of lipodystrophy are sparse. In addition to one previously reported case in FPLD1, RYGB has been used in FPLD2 (*LMNA*) and FPLD4 [perlipin 1 (*PLIN1*)] monogenic lipodystrophies (6–10). In our FPLD1 cases, RYGB achieved glycemic targets where optimum medical therapy had been unsuccessful, additionally reducing ectopic fat deposition as a likely critical mediator of the metabolic dysregulation observed in this condition. RYGB may be an important adjunct to optimal medical management of patients with FPLD1.
