# Supplementary material for: Roux-en-Y Gastric Bypass Surgery in the Management of Familial Partial Lipodystrophy Type 1
Source: J Clin Endocrinol Metab. 2017 Jul 26;102(10):3616–20. doi: 10.1210/jc.2017-01235 (PMC5630252; doi:10.1210/jc.2017-01235)
Supplement: Supplementary file 1 [file jc.2017-01235.sf1.pdf]

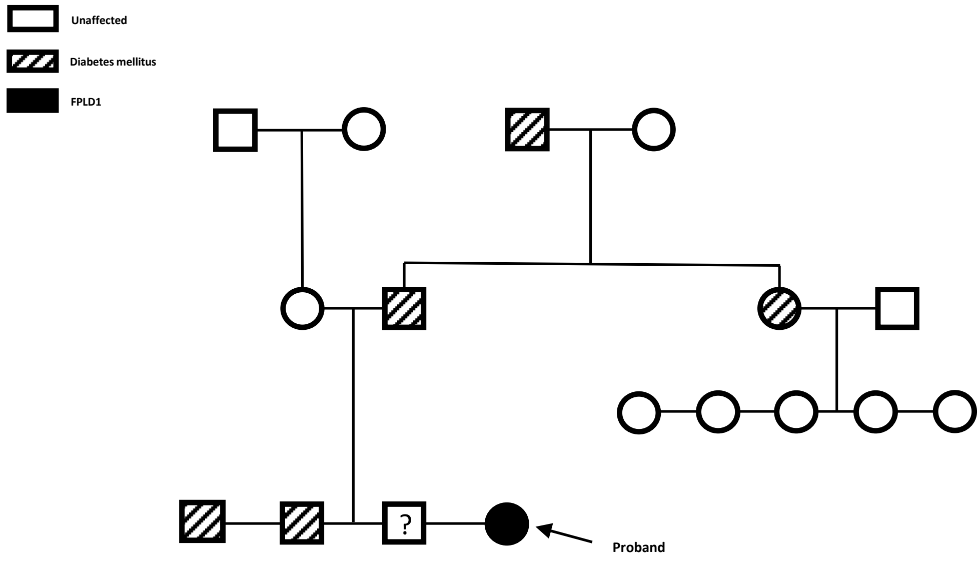

Supplemental Figure 1: Pedigree for patient A

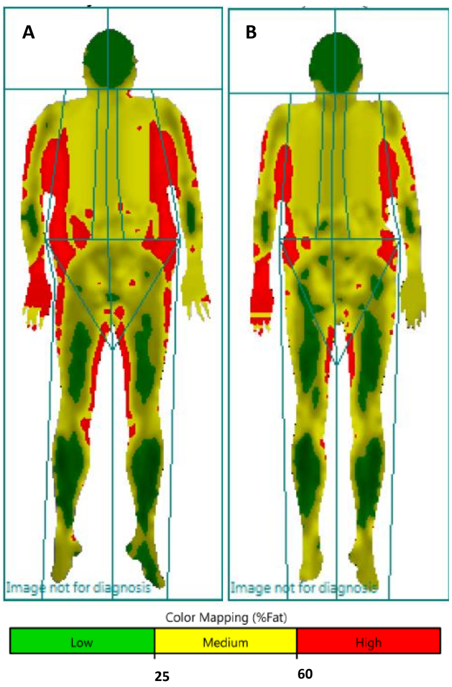

Supplemental Figure 2: DEXA images of patient A demonstrating the distribution of body fat in FPLD1. **Image A** shows the selective distribution of fat in the trunk and abdominal region and a paucity of fat in extremities at one month pre-operatively. **Image B** illustrates the reduction in body fat at 3 months following RYGB. Increased colour mapping of the right forearm and hand represent artefact due to cannulation.

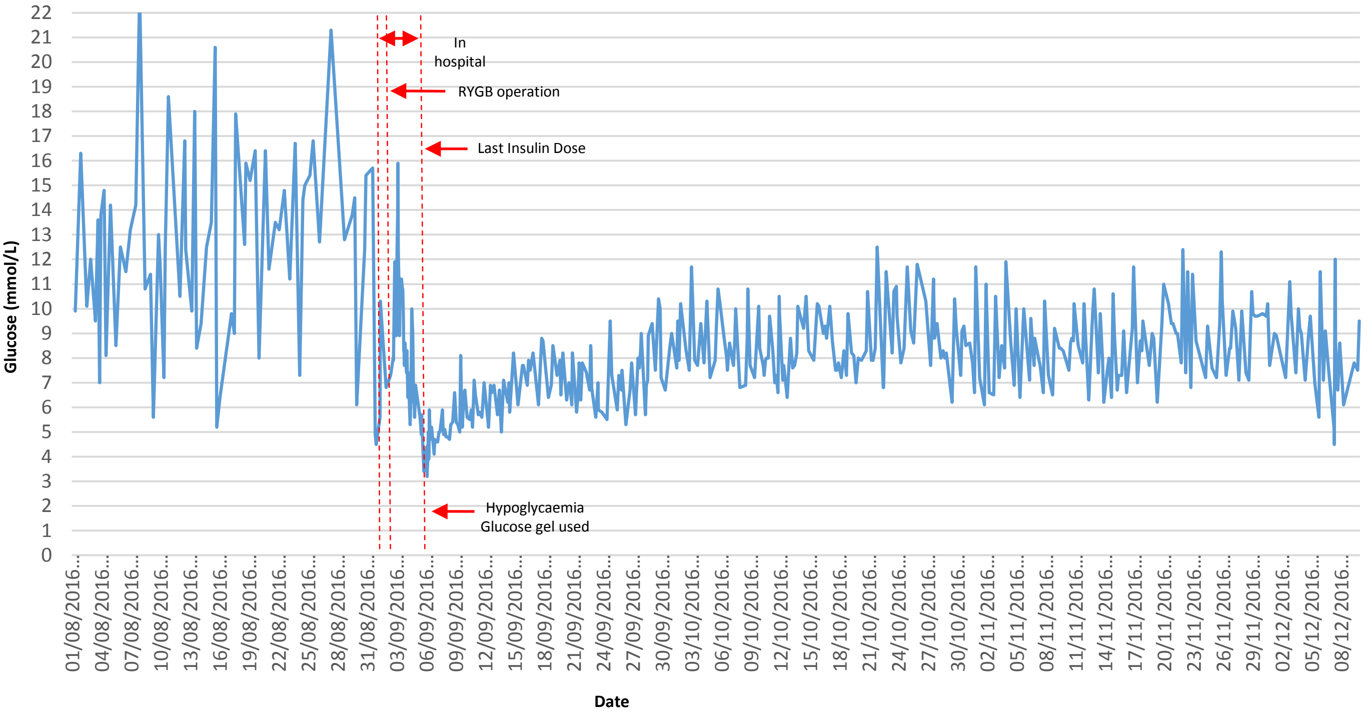

Supplemental Figure 3: Patient A self-recorded capillary blood glucose levels during the pre and post-operative period

Example menu plan

(1000kcal /day. 40-50grams carbohydrate per meal)

Breakfast

150g low sugar beans with one slice of medium sliced bread (no butter or spread)

Or

50grams porridge oats made with 150ml skimmed milk

Snack

(Choose one)

Small piece of fruit or handful of berry sized fruit.

Lunch

Two thinly cut sliced wholemeal bread with thinly sliced chicken breast (100g) and salad. Use thin scraping of low fat spread or low fat salad cream.

One medium piece of fruit

One low fat yoghurt (100-120g pot less than 100Kcal)

Or

250g bowl of vegetable based soup and 1 slice of thinly sliced wholemeal bread (no butter / spread)

Snack

Vegetable sticks, such as cucumber and carrots with tomato salsa

Evening meal

Spaghetti bolognaise made with meat alternative such as 75g Quorn mince ® (recipe as per diet sheet)

Supplemental Figure 4: Sample dietary advice for a patient with FPLD1
